# Ovarian Cancer With Breast Metastasis and Two Pathogenic Variants of BRCA1 Gene

**DOI:** 10.7759/cureus.18691

**Published:** 2021-10-11

**Authors:** Saeedeh Kowsarnia, Nader Javadi

**Affiliations:** 1 Research, Olive View-University of California, Los Angeles (UCLA) Education & Research Institute, Sylmar, USA; 2 Oncology, Hope Health Center, Calabasas, USA

**Keywords:** ovarian cancer treatment, metastatic ovarian cancer, immuno-chemotherapy, target therapy oncology, brca1, precision cancer medicine, breast metastasis, ovarian tumors, next generation sequencing (ngs), brca gene mutation

## Abstract

Ovarian cancer is the second most common gynecologic cancer after uterine cancer in the United States. Ovarian cancer ranks sixth in cancer deaths among women, accounting for more deaths than other female reproductive system cancers. Breast metastasis in ovarian cancer is a rare presentation and predicts a poor prognosis and challenging management. Our case is a 42-year-old Chinese woman with high-grade serous ovarian carcinoma that presents with metastasis to the breast during the course of her illness. Genetic evaluation of the ovarian tumor showed two BRCA1 pathogenic variants. Germline pathogenic variant of c.2110_2111DelAA and a somatic variant of c.4071_4096+14del40. Our patient was offered different treatment regimens but showed progression of her disease. The low survival rate and high recurrence rate in ovarian cancer show that we still need to investigate our current approved treatments. Our report aims to shed light on the genetic evaluation of ovarian tumors and treatment options available in refractory cases of progressive ovarian cancer. Furthermore, we explain our investigational therapy regimen and the reasoning behind it.

## Introduction

Ovarian cancer is the second most common gynecologic cancer after uterine cancer in the United States. Ovarian cancer ranks sixth in cancer deaths among women, accounting for more deaths than other female reproductive system cancers [1,2]. In Women, the risk of getting ovarian cancer during their lifetime is about 1 in 78, and the lifetime chance of dying from ovarian cancer is about 1 in 108 [3]. It is estimated that 75% of patients present with advanced stage, with either widely metastatic disease within the peritoneal cavity or distant metastasis [4]. Ovarian cancers rarely metastasize to the breast and axillary lymph nodes [5,6]. The differentiation between the primary breast neoplasm from metastasis is required for a proper approach to treatment since the treatment and prognosis are quite different. BRCA1 protein has a crucial role in several pathways to maintain DNA integrity through DNA repair [7]. Loss of BRCA1 function due to the mutation causes genomic instability resulting in oncogenic transformation and cancer evolution [8]. Germline BRCA1 pathogenic variants are associated with early-onset breast and ovarian cancers [9-11]. Studies showed that only 18% of the cases of ovarian cancers have germline mutations [12]. It is estimated that 3% of the patients have somatic BRCA1/2 gene mutations in the specimens [9]. Different studies elaborated upon diverse variants of pathogenic BRCA1 gene variants in populations worldwide [13]. The survival rate for ovarian cancers is relative to the stage. For stage III C, the five-year survival rate is 39% [14]. The high risk of recurrence in ovarian cancers mandates investigating a new treatment regimen. In this case report, we present a case of ovarian cancer with germline and somatic pathogenic variants of the BRCA1 gene who presented with breast metastases during the course of her disease. We discuss the genetic aspect of the case and the current treatment available for recurrent, progressive ovarian cancer comparing to our investigational treatment regimen.

## Case presentation

Our case is a 42-year-old Chinese female who was 38-year-old and premenopausal at the time of the diagnosis. Our case complained of nausea and vague lower abdominal pain, and heaviness for months before the diagnosis. Primarily, she presented with a nodule of 3×1×2 cm in the umbilical region, penetrating outward through umbilical skin. Primary Ultrasound showed multiple heterogeneous masses occupying both adnexa. CT scan of the abdomen and pelvic in October 2015 showed complex cystic/solid pelvic mass in both adnexa and subcapsular nodules at the right side of the liver.

In November 2015, the patient underwent an exploratory laparotomy. The surgical evaluation revealed involvement of both adnexa, 5×5×4cm tumor on the right side and 3×2.5×1.5cm tumor on the left side. Several cauliflower-like tumors are scattered throughout in peritoneum. Radical resection, including hysterectomy, bilateral salpingo-oophorectomies, resection of the greater omentum, overlying liver masses, a tumor from septum transversum, pelvic lymph nodes, and umbilical mass was performed. Repair of intestine and rectum and bladder with lysis of intestinal adhesion was conducted in the same procedure. Microscopic evaluation of resected tissue with immunohistochemistry (IHC) and surgical evaluation confirmed the diagnosis as high-grade serous carcinoma of bilateral ovaries (HGSOC), FIGO (International federation of gynecology and obstetrics) stage III C.

Germline genetic assay with CIRCULOGENE revealed NM _007294 BRCA1 frameshift mutation c.2110_2111DelAA at DNA level, highly pathogenic and p.Asn704Cysfs*7 at the product level. Our patient was a heterozygous carrier for c.2110_2111delAA BRCA1 Mutation. Genetic examination on surgical tissue on umbilicus mass showed additional oncogenic mutations; TP53 c.1025G>C p.R342P and NOTCH2 c.3930G> p.Q1310H.

The next-generation sequencing assay on ovarian tissue detected additional somatic BRCA1 pathogenic gene variants. This variant is deletion mutation c.4071_4096+14del40, and the product is the Intron splice variant. Biomarkers evaluation showed microsatellite instability (MSI-stable) with TMB (Tumor Mutational Burden) 11 mutation/MB (Intermediate). Immunochemistry studies showed PR (progesterone receptor) and ER (estrogen receptor) positive, PDL-1 negative (programmed death ligand-1).

Bilateral breast mammography at the time of diagnosis was unremarkable. Our case had a history of a right ovarian cystectomy in 2012 with pathology reported a simple cyst. Our case had been conceived once with the help of IVF (in vitro fertilization) due to infertility. She had a cesarean section for a male infant in 2014, with the inspection of ovaries showed no abnormalities. Family history was positive for ovarian cancer in mother who deceased at 48 years of age with unknown BRCA status. Initial tumor markers at the time of diagnosis in October 2015 showed CEA= 0.71, AFP= 1.79, CA 19-9= 7.87, CA 125= 131.40, CA 72-4= 7.97, HCG =< 1.20, SCC= 0.88, CA (HE-4) =180.40.

The patient received different cycles of chemotherapy between November 2015 to March 2019. She was initially treated with Carboplatin and Taxol. She was partially platinum-sensitive since she had disease recurrence within seven months of chemotherapy with Carboplatin and Taxol. She participated in three different clinical trials under the surveillance of CT scan imaging every 2-3 months. First trial PARP inhibitor with mTORC1/2 inhibitor or oral AKT inhibitors. According to RECIST1.1 (Response Evaluation Criteria in Solid Tumors) criteria, the patient showed disease progression. The level of CA 125 was progressively high in all three trials. After the first trial, She received Carboplatin and Doxil for six cycles. New tumor growth was observed in the Vaginal vault causing bowel obstruction requiring surgical intervention. She attended the second trial, phase II ATR Inhibitor. Due to the tumor progression, She was withdrawn from the 2nd trial after a month to receive Taxol and Avastin for six cycles from September 2018 to March 2019. In March 2019, she presented with widespread involvement of diaphragmatic and para-aortic lymph nodes with a CA 125 level of nearly 1114 U/ml. In April 2019, She participated in the third trial to receive Olaparib but was advised to another treatment regimen, Topotecan, and Bevacizumab, after a month. CT scan in May 2018 showed a new prominent left supraclavicular lymph node measured 4x7mm, with progression to 7x9 mm in Dec 2018 and 8x13 mm in March 2019. In May 2019, mildly prominent new left axillary and right hilar lymph nodes were noted.

**Figure 1:**
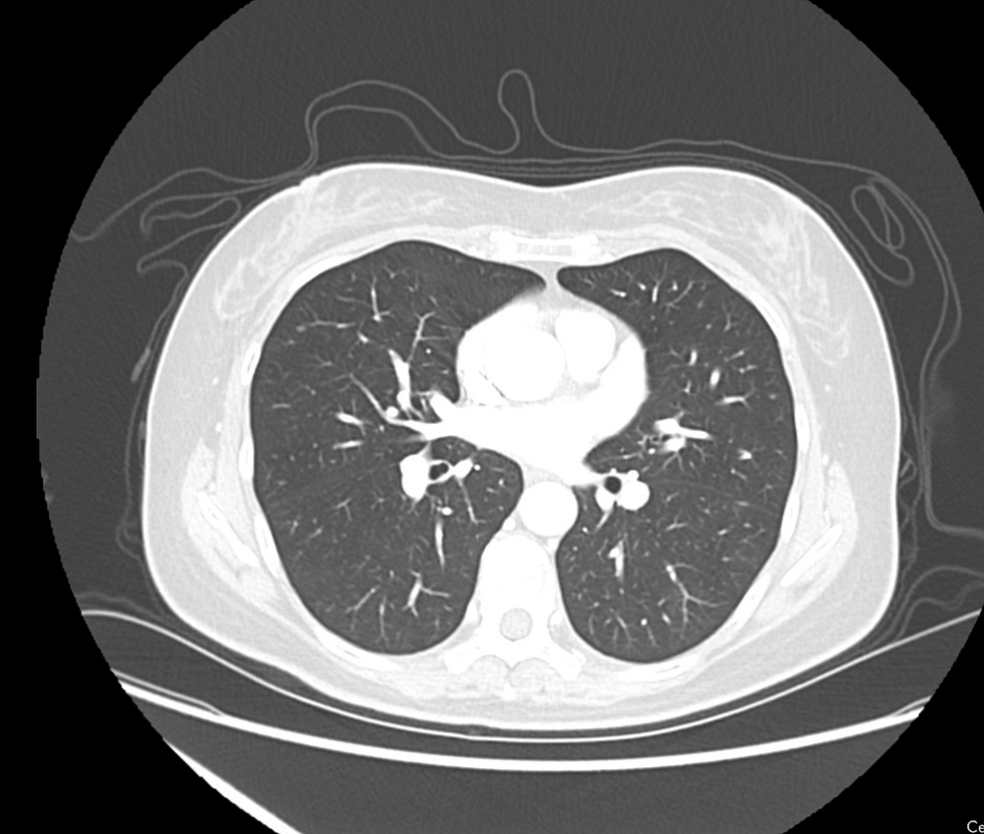
Chest CT scan shows an ill-defined asymmetrical mass in the medial aspect of the left breast.

**Figure 2:**
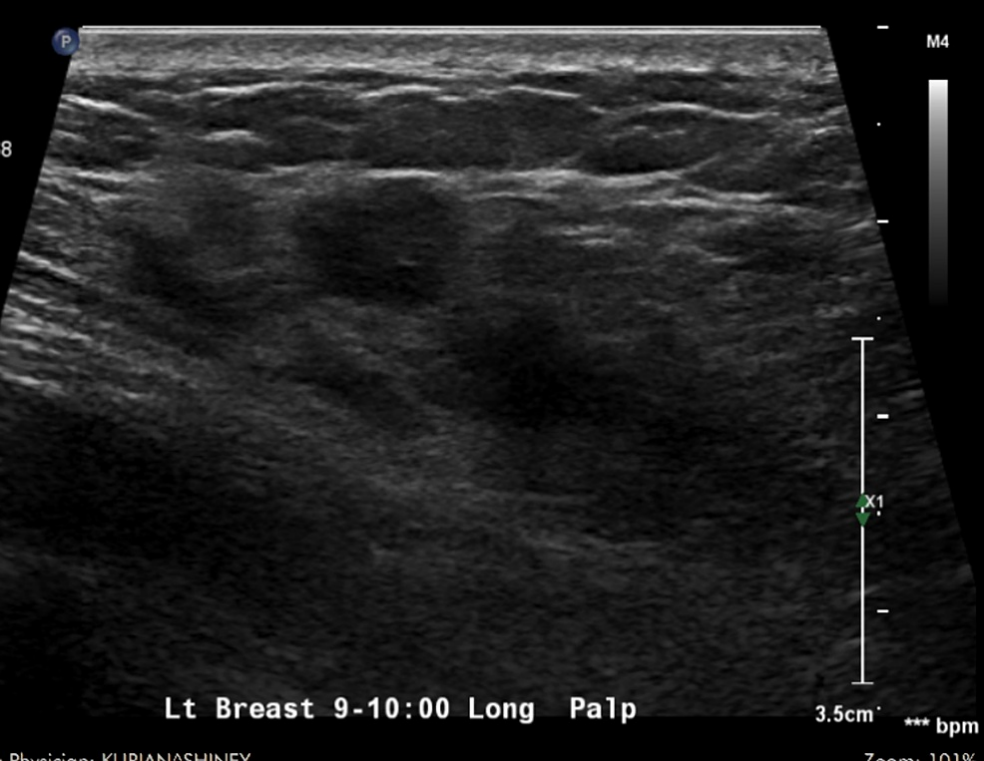
Left breast real-time US shows hypoechoic coalescing innumerable masses 6x3.5x1.5 cm in the longitudinal plane. Subtle hypoechoic mass 2.5x2.3x0.6 cm in left pectoralis muscle.

In June 2019, she presented with vague breast discomfort and a lump in the left breast, positioned at 9-o’clock and 4 cm from the nipple upper inner quadrant. CA 125 level was 2060 U/ml. Chest CT scan showed asymmetrical masses in the medial aspect of the left breast (Figure 1). Left Chest US (ultrasound) in grayscale showed lobulated hypoechoic mass 4x1.4 in the longitudinal plane with subtle hypoechoic mass measuring 1.9x0.7 cm seen in left pectoralis muscle. The real-time US of the left breast revealed irregular hypoechoic coalescing innumerable masses from 8 to 12 o’clock position in the left breast measured at least 6x 3.5x1.5 cm collectively (Figure 2). There was a 2.5x2.3x0.6 cm hypoechoic mass in the underlying left pectoralis muscle deep to the 9-o’clock position of the left breast. The mammogram showed focal heterogenous asymmetry 6x4x2 cm in 8-11 o’clock position, 4 cm from the nipple. Core needle-guided biopsy of the left breast mass was performed. Pathology report demonstrated infiltrative carcinoma with the micropapillary pattern associated with microcalcifications. The immunohistochemical staining of the breast specimen was diffusely positive for PAX8, WT-1 and negative for GATA3. Wild-type TP53 was also detected. All data were consistent with metastatic serous carcinoma of the ovary.

**Figure 3:**
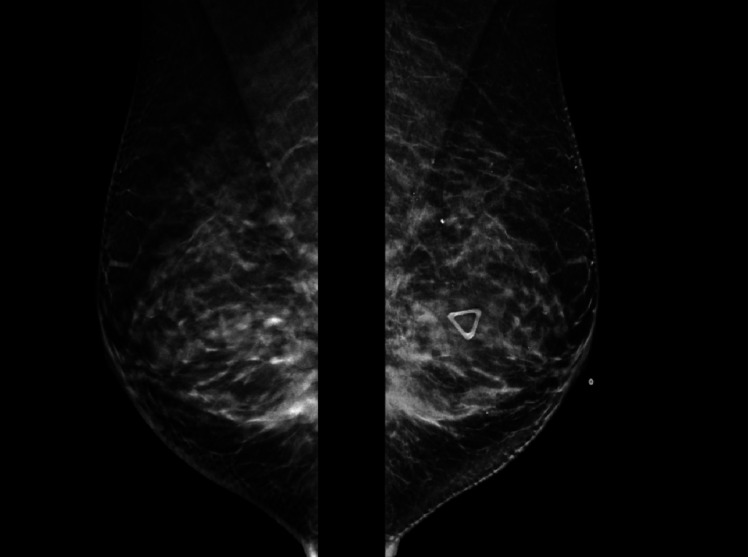
Breast tomosynthesis mammogram of the left breast shows defined sets of calcification. Left is L MLO view (Left Mediolateral oblique), Right is mediolateral oblique caudal view.

Breast tomosynthesis in Figure 3 shows Focal heterogenous asymmetry with scattered calcification. She was referred to us from hospice care as she had demanded to evaluate further treatment options. She had massive ascites, generalized weakness, and abdominal pain with CA 125 was 6251 U/ml. Our investigational 8th line treatment consisted of a combination of chemotherapy and immunotherapy. A reduced dose of Pemetrexed with Nab-PAClitaxel every three weeks. Immunotherapy with nivolumab every two weeks, and targeted therapy with Temsirolimus, Ramucirumab every three weeks. After the 2nd month of treatment, her performance status was significantly improved from ECOG (Eastern Cooperative Oncology Group) performance status 4 to 2. She had significant improvement in energy level. After the 4th cycle of the treatment, abdominal distention was resolved, and the CA 125 level decreased to 493 U/ml. Unfortunately, the patient presented with nausea, vomiting, and severe abdominal pain. Abdominal Imaging showed pneumoperitoneum requiring hospital admission and possible surgery. She had previous abdominal surgery to relieve bowel obstruction. She refused further treatment in the hospital and passed away due to the consequences of sepsis.

## Discussion

In women, epithelial cancers can originate from the ovaries, fallopian tubes, and peritoneal cells, with ovarian cancers consist of 90% of all epithelial tumors in the abdominal cavity [15]. Approximately 15% of ovarian cancer is localized to the ovary, 17% is regional, and 62% manifests as a distant disease [16]. The most common sites of distant metastasis in ovarian cancers are the liver, followed by distant lymph nodes, lung, bone, and brain, respectively [17]. Ovarian cancers rarely metastasize to the breast [5]. Metastasis to the breast consists of only 2% of all breast malignancies, with ovarian cancer accounting for only 0.03 to 0.6% [18]. The most common primary site of metastasis to the breast is the contralateral primary breast tumor, followed by lung and ovary [19]. Metastatic breast cancers from gynecological tumors are very rare, with an overall incidence of 0.17%, with the ovaries as the origin of metastases in 0.07% of all cases [20,21]. Breast involvement in the course of ovarian cancer is associated with a poor prognosis [19].

Caruso et al. reported 120 cases of ovarian cancer with breast metastasis between 1999 to 2019. Serous carcinoma of the ovaries is the most common histologic type of ovarian cancer metastases to the breast [5]. It was reported that there was a preference for metastasis to the left breast [5,22]. The ascites are drained through the lymph nodes into the thoracic duct that eventually drains into the left subclavian vein and blood circulation; thus, the role of the hematogenous route is more prominent.

Bitencourt et al. investigated radiological findings in breast metastasis from extramammary origins. They concluded that breast metastasis generally has microlobulated or indistinct margins. No spiculated margins, skin, or nipple retraction is usually seen. Calcifications are not frequently present in metastatic lesions; however, they occur more commonly in patients with ovarian cancer [23]. Hematogenous spread lesions, similar to our case, tend to be more circumscribed in contrast to lymphatic spread, which leads to diffuse involvement with edema and thickening of the skin [24]. Moreover, the most common tumor site was the upper outer quadrant; contrary to our case, the mass was located in the upper inner quadrant [25].

Hence the radiologic features of metastatic breast lesions are variable; the backbone of the diagnosis in metastatic breast lesions is a core needle biopsy. Immunohistochemical (IHC) stains help differentiate between the primary breast lesions and metastatic lesions. PAX 8 and WT-1 are transcriptional factors abundantly seen in female genital tract pathologies [26]. Another characteristic of HGSOC is the detection of aberrant TP53 in the pathology using IHC [27].

Germline genetic assay revealed pathogenic BRCA1 gene germline mutation c.2110_2111delAA (2229delAA). Recent case reports showed the association of BRCA1 mutation c.2110_2111delAA with invasive breast ductal carcinoma with ER, PR, and HER2 negative in two middle-aged Chinese females. Both cases had a familial history of breast and early-onset ovarian cancer in their families [28]. Kim YC et al. performed an extensive literature review to identify all BRCA1 mutations in the Chinese population. From 2003 to 2015, 822 cases of c.2110_2111delAA mutation were reported with a 1% carrier rate [29]. This c.2110_2111delAA mutation is a frameshift mutation that creates the premature translational stop in the BRCA1 gene causes absent or disrupted protein products. As Chen L. et al. depicted in their study, this mutation is a frameshift mutation with deletion of 2 Adenosine bases resulting in a stop codon in the translation process of the BRCA1 gene. The product protein is named p.Asn704Cysfs*7 [30]. No studies compared the treatment response and clinicopathological effect of different variants of BRCA1 gene mutations in patients with ovarian cancer.

Next-generation sequencing on the breast tissue showed another variant of BRCA1 mutation c.4071_4096+14del40. The gene frequency was consistent with somatic mutation. We could not find any literature reporting this variant of BRCA1 transformation. Variant c.4071_4096+14del40 is a 14 nucleotide deletion causing an intron to be translated into the BRCA1 protein and is considered highly pathogenic.

Kim et al. reported that in advanced-stage high-grade serous ovarian cancer, patients with germline BRCA1/2 mutations have a better prognosis with more prolonged progression-free survival than those lacking BRCA mutations [31]. The cases with BRCA1 germline mutations tend to be younger than the wild-type BRCA1 cases in the same study. However, there was no study to compare outcome differences among the variants of BRCA1 mutations in patients with ovarian cancer.

Partially platinum-sensitive ovarian cancer was defined as the PFI (Platinum free interval) in 6 to 12 months after the last dose of platinum-based therapy in ovarian cancer patients. Platinum-sensitive ovarian cancer has a median survival of two years, ranging from three months to over 10 years. Platinum-resistant ovarian cancer has a 9-12 months median survival, with less than 15% responding to successive chemotherapy. Ultimately, almost all HGSOC patients become platinum-resistant. Among the subtype of ovarian cancers, high-grade serous carcinoma was the most responsive to platinum-based chemotherapy [32]. In conclusion to all the studies regarding the mechanism underlying platinum-resistant ovarian cancer, the genetic mechanism that causes genomic instability causes the tumor cells to adapt and survive the DNA damage by chemotherapeutic agents and escape apoptosis. Our patient has a variant of BRCA1 mutation that impede the BRCA1 translation, resulting in absent BRCA1 protein with impairment of apoptosis signals associated with BRCA1.

TP53 c.1025G>C mutation was detected in the breast tissue and metastatic masses in our case’s abdominal wall and inguinal area. TP53 c.1025G>C mutation was one of the pathogenic missense mutations resulting in an inactive TP53 protein, defective at both tetramerization and transcriptional transactivation [33]. Boyarskikh et al. investigated the different types of TP53 somatic mutations associated with ovarian carcinomas with BRCA1/2 germline mutations. They observed that somatic TP53 mutation variants were expressed in 100% of HGSOC (high-grade serous ovarian cancer) cases, with the missense type defined as the most common variant. Due to the role of the TP53, the variants will be detected in all the tumor cells and subclones in metastasis during the treatment [34]. Studies showed that HGSOC carrying concurrent somatic variants of TP53 had worse outcome indices [35]. Tuna et al. reviewed different TP53 hotspot mutations in 791 HGSOC patients. They concluded that individual TP53 hotspot mutations have a different impact on HGSOC patients’ outcomes and response to therapy. They proposed the status of TP53 modifications could help to select effective treatment regimens [36].

First-line treatment in ovarian cancer is a platinum agent with a Taxane [37]. The anti-angiogenic agent, Bevacizumab, is approved by the food and drug administration(FDA) for both frontline and maintenance therapy in ovarian cancer [38]. FDA approved the combination of Olaparib and Bevacizumab for maintenance therapy in patients with BRCA mutations ovarian cancer who responded to the first-line treatment [39,40]. A platinum-based combination treatment regimen is still preferred in patients with recurrent disease [41]. The ideal combination of platinum-based chemotherapy is still unknown. Maintenance therapy is recommended for all HGSOC as patients on maintenance therapy showed a better response to future treatments.

Ramucirumab, a humanized monoclonal antibody that targets vascular endothelial growth factor receptor-2 (VEGFR2), has also increased overall and progressive free survival in patients with advanced solid tumors [42]. Although, in another study, monotherapy with anti-angiogenic agents has not shown any survival benefits in patients with ovarian cancer [43].

Checkpoint inhibitor immunotherapy, Nivolumab, is a monoclonal human IgG4 antibody against programmed death 1 (PD-1) ligand. Studies have shown the maximum treatment response in cases with low and high PD-L1 expression levels; therefore, Investigators do not recommend having PD-L1 biomarkers as an identifier of eligibility for immunotherapy treatment [44]. New data confirmed that even a monotherapy with single immune checkpoint inhibitors resulted in a higher overall survival rate, progression-free survival, and higher overall response rate compared to platinum-based chemotherapy in solid tumors [45]. A growing body of evidence discusses the synergistic effect of checkpoint inhibitors and anti-angiogenic agents in treating solid tumors [46].

Recent review still suggested pemetrexed, alone or in combination, in salvage therapy in ovarian cancers unresponsive to the other lines of treatment [47]. Pemetrexed is a folate antagonist of α-receptors and antineoplastic agent used in treating various tumors [48]. The combination of Pemetrexed and Bevacizumab showed disease control in 94% of the cases in platinum-resistant and platinum-sensitive recurrent ovarian cancers [49]. The ongoing clinical trials assessing the benefit of folate receptors antagonist in the diagnosis and treatment of cancer patients are evidence of the capability of these agents in improving the future care of cancer patients [48].

PI3K/AKT/mTOR is one of the most investigated intracellular signaling pathways. In several tumors, including ovarian cancer, dis-regulation of this pathway was observed [50,51]. Temsirolimus, an mTOR inhibitor, showed tumor stabilization in patients with progressive ovarian tumors with minimum toxicity [52]. New Studies with a new generation of mTOR inhibitors alone or in combination showed promising results in treating ovarian cancer [53,54].

There are different molecular pathways recognized in ovarian cancer evolution [55]. Our case had recurrent, progressive disease unresponsive to the suggested current lines of treatment. We recommended our investigational treatment regimen, according to the published data regarding the effectiveness of the agents against various oncogenic pathways involved in ovarian cancers. This regimen was not caused any side effects or complications for the patient.

## Conclusions

In patients with the BRCA1 gene mutation, annual breast evaluation is mandatory for the early detection of breast pathologies. Despite the rarity of breast metastasis from ovarian cancers, In patients with ovarian cancer and BRCA1 pathogenic gene variants, the possibility of metastasis from ovarian cancer should always be considered in the presence of the axillary lymph node enlargement. Metastatic ovarian cancer to the breast is a rare presentation and associated with a poor prognosis. IHC methods are the backbone of the diagnosis of metastatic breast lesions. Any new distant metastasis in the course of ovarian cancer could be associated with chemoresistance status.

Higher mortality rate and lower survival rate mandate assessment of the new treatment options in HGSOC. Individualized treatment according to each patient’s molecular and genetic properties could be a promising step in discovering new treatment options for patients with HGSOC. Further investigations of the genetic and molecular details in HGSOC could be helpful to evaluate the causes of chemotherapy resistance in patients with progressive disease. Current effective combination therapies in other solid tumors could be an alternative line of treatment in HGSOC patients who are not responsive to the current recommendations. Either Checkpoint inhibitors, mTOR inhibitors, antifolate receptor inhibitors, angiogenesis inhibitors, alone or in combination, can constitute effective regimens for patients with progressive disease who failed recommended lines of treatment. Future clinical trials to investigate the survival benefit and possible side effects may warrant these agents in HGSOC treatment.

## References

[1] (2021). Cancer Statistics. https://www.cancer.org/cancer/ovarian-cancer/about/key-statistics.html.

[2] (2021). Ovarian Cancer Statistics. https://www.cdc.gov/cancer/ovarian/statistics/index.htm.

[3] (2021). Key Statistics for Ovarian Cancer. https://www.cancer.org/cancer/ovarian-cancer/about/key-statistics.html.

[4] Lengyel E (2010). Ovarian cancer development and metastasis. Am J Pathol.

[5] Caruso G, Musacchio L, Santangelo G (2020). Ovarian cancer metastasis to the breast: a case report and review of the literature. Case Rep Oncol.

[6] Recine MA, Deavers MT, Middleton LP, Silva EG, Malpica A (2004). Serous carcinoma of the ovary and peritoneum with metastases to the breast and axillary lymph nodes: a potential pitfall. Am J Surg Pathol.

[7] Gudmundsdottir K, Ashworth A (2006). The roles of BRCA1 and BRCA2 and associated proteins in the maintenance of genomic stability. Oncogene.

[8] Peitzsch C, Tyutyunnykova A, Pantel K, Dubrovska A (2017). Cancer stem cells: the root of tumor recurrence and metastases. Semin Cancer Biol.

[9] (2011). Integrated genomic analyses of ovarian carcinoma. Nature.

[10] Rosen EM, Fan S, Pestell RG, Goldberg ID (2003). BRCA1 gene in breast cancer. J Cell Physiol.

[11] Neff RT, Senter L, Salani R (2017). BRCA mutation in ovarian cancer: testing, implications and treatment considerations. Ther Adv Med Oncol.

[12] Frey MK, Pothuri B (2017). Homologous recombination deficiency (HRD) testing in ovarian cancer clinical practice: a review of the literature. Gynecol Oncol Res Pract.

[13] Karami F, Mehdipour P (2013). A comprehensive focus on global spectrum of BRCA1 and BRCA2 mutations in breast cancer. Biomed Res Int.

[14] Survival Rates for Ovarian Cancer. (2021). Accessed. https://www.cancer.org/cancer/ovarian-cancer/detection-diagnosis-staging/survival-rates.html..

[15] Heintz AP, Odicino F, Maisonneuve P (2006). Carcinoma of the ovary. FIGO 26th Annual Report on the Results of Treatment in Gynecological Cancer. Int J Gynaecol Obstet.

[16] Jelovac D, Armstrong DK (2011). Recent progress in the diagnosis and treatment of ovarian cancer. CA Cancer J Clin.

[17] Deng K, Yang C, Tan Q (2018). Sites of distant metastases and overall survival in ovarian cancer: a study of 1481 patients. Gynecol Oncol.

[18] Georgiannos SN, Chin J, Goode AW, Sheaff M (2001). Secondary neoplasms of the breast: a survey of the 20th century. Cancer.

[19] Klein RL, Brown AR, Gomez-Castro CM, Chambers SK, Cragun JM, Grasso-LeBeau L, Lang JE (2010). Ovarian cancer metastatic to the breast presenting as inflammatory breast cancer: a case report and literature review. J Cancer.

[20] Hajdu SI, Urban JA (1972). Cancers metastatic to the breast. Cancer.

[21] DeLair DF, Corben AD, Catalano JP, Vallejo CE, Brogi E, Tan LK (2013). Non-mammary metastases to the breast and axilla: a study of 85 cases. Mod Pathol.

[22] Lee AH (2007). The histological diagnosis of metastases to the breast from extramammary malignancies. J Clin Pathol.

[23] Bitencourt AG, Gama RR, Graziano L (2017). Breast metastases from extramammary malignancies: multimodality imaging aspects. Br J Radiol.

[24] Sippo DA, Kulkarni K, Di Carlo P (2016). Metastatic disease to the breast from extramammary malignancies: a multimodality pictorial review. Curr Probl Diagn Radiol.

[25] Moore DH, Wilson DK, Hurteau JA (1998). Gynecologic cancers metastatic to the breast. J Am Coll Surg.

[26] Liliac L, Carcangiu ML, Canevari S (2013). The value of PAX8 and WT1 molecules in ovarian cancer diagnosis. Rom J Morphol Embryol.

[27] Cole AJ, Dwight T, Gill AJ (2016). Assessing mutant p53 in primary high-grade serous ovarian cancer using immunohistochemistry and massively parallel sequencing. Sci Rep.

[28] Cao WM, Zheng YB, Gao Y (2019). Comprehensive mutation detection of BRCA1/2 genes reveals large genomic rearrangements contribute to hereditary breast and ovarian cancer in Chinese women. BMC Cancer.

[29] Kim YC, Zhao L, Zhang H (2016). Prevalence and spectrum of BRCA germline variants in mainland Chinese familial breast and ovarian cancer patients. Oncotarget.

[30] Chen L, Fu F, Huang M, Lv J, Zhang W, Wang C (2020). The spectrum of BRCA1 and BRCA2 mutations and clinicopathological characteristics in Chinese women with early-onset breast cancer. Breast Cancer Res Treat.

[31] Kim SI, Lee M, Kim HS, Chung HH, Kim JW, Park NH, Song YS (2019). Effect of BRCA mutational status on survival outcome in advanced-stage high-grade serous ovarian cancer. J Ovarian Res.

[32] Davis A, Tinker AV, Friedlander M (2014). "Platinum resistant" ovarian cancer: what is it, who to treat and how to measure benefit?. Gynecol Oncol.

[33] Kamada R, Nomura T, Anderson CW, Sakaguchi K (2011). Cancer-associated p53 tetramerization domain mutants: quantitative analysis reveals a low threshold for tumor suppressor inactivation. J Biol Chem.

[34] Boyarskikh UA, Gulyaeva LF, Avdalyan AM (2020). Spectrum of TP53 mutations in BRCA1/2 associated high-grade serous ovarian cancer. Front Oncol.

[35] Lassus H, Leminen A, Lundin J, Lehtovirta P, Butzow R (2003). Distinct subtypes of serous ovarian carcinoma identified by p53 determination. Gynecol Oncol.

[36] Tuna M, Ju Z, Yoshihara K, Amos CI, Tanyi JL, Mills GB (2020). Clinical relevance of TP53 hotspot mutations in high-grade serous ovarian cancers. Br J Cancer.

[37] Kyrgiou M, Salanti G, Pavlidis N, Paraskevaidis E, Ioannidis JP (2006). Survival benefits with diverse chemotherapy regimens for ovarian cancer: meta-analysis of multiple treatments. J Natl Cancer Inst.

[38] Haunschild CE, Tewari KS (2020). Bevacizumab use in the frontline, maintenance and recurrent settings for ovarian cancer. Future Oncol.

[39] Domchek SM, Aghajanian C, Shapira-Frommer R (2016). Efficacy and safety of olaparib monotherapy in germline BRCA1/2 mutation carriers with advanced ovarian cancer and three or more lines of prior therapy. Gynecol Oncol.

[40] Arora S, Balasubramaniam S, Zhang H (2021). FDA approval summary: olaparib monotherapy or in combination with bevacizumab for the maintenance treatment of patients with advanced ovarian cancer. Oncologist.

[41] Pignata S, Scambia G, Bologna A (2017). Randomized controlled trial testing the efficacy of platinum-free interval prolongation in advanced ovarian cancer: the MITO-8, MaNGO, BGOG-Ov1, AGO-Ovar2.16, ENGOT-Ov1, GCIG Study. J Clin Oncol.

[42] Wang K, Qu X, Wang Y (2016). The impact of ramucirumab on survival in patients with advanced solid tumors: a systematic review and meta-analysis of randomized II/III controlled trials. Clin Drug Investig.

[43] Penson RT, Moore KM, Fleming GF (2014). A phase II study of ramucirumab (IMC-1121B) in the treatment of persistent or recurrent epithelial ovarian, fallopian tube or primary peritoneal carcinoma. Gynecol Oncol.

[44] Wang C, Qiao W, Jiang Y (2020). The landscape of immune checkpoint inhibitor plus chemotherapy versus immunotherapy for advanced non-small-cell lung cancer: a systematic review and meta-analysis. J Cell Physiol.

[45] Ferrara R, Imbimbo M, Malouf R, Paget-Bailly S, Calais F, Marchal C, Westeel V (2020). Single or combined immune checkpoint inhibitors compared to first-line platinum-based chemotherapy with or without bevacizumab for people with advanced non-small cell lung cancer. Cochrane Database Syst Rev.

[46] Manegold C, Dingemans AC, Gray JE (2017). The potential of combined immunotherapy and antiangiogenesis for the synergistic treatment of advanced NSCLC. J Thorac Oncol.

[47] Dottino P, Dashkoff M, Beddoe AM (2020). Feasibility of using pemetrexed as a salvage regimen in heavily pre-treated patients with ovarian cancer: a retrospective review. Indian J Cancer.

[48] Scaranti M, Cojocaru E, Banerjee S, Banerji U (2020). Exploiting the folate receptor α in oncology. Nat Rev Clin Oncol.

[49] Hagemann AR, Novetsky AP, Zighelboim I (2013). Phase II study of bevacizumab and pemetrexed for recurrent or persistent epithelial ovarian, fallopian tube or primary peritoneal cancer. Gynecol Oncol.

[50] Gasparri ML, Bardhi E, Ruscito I (2017). PI3K/AKT/mTOR pathway in ovarian cancer treatment: are we on the right track?. Geburtshilfe Frauenheilkd.

[51] Weng H, Feng X, Lan Y, Zheng Z (2021). TCP1 regulates PI3K/AKT/mTOR signaling pathway to promote proliferation of ovarian cancer cells. J Ovarian Res.

[52] Emons G, Kurzeder C, Schmalfeldt B (2016). Temsirolimus in women with platinum-refractory/resistant ovarian cancer or advanced/recurrent endometrial carcinoma. A phase II study of the AGO-study group (AGO-GYN8). Gynecol Oncol.

[53] Tran AQ, Sullivan SA, Chan LL (2020). SPR965, a dual PI3K/mTOR inhibitor, as a targeted therapy in ovarian cancer. Front Oncol.

[54] Jacome Sanz D, Raivola J, Karvonen H, Arjama M, Barker H, Murumägi A, Ungureanu D (2021). Evaluating targeted therapies in ovarian cancer metabolism: novel role for PCSK9 and second generation mTOR inhibitors. Cancers.

[55] Liu CL, Yuan RH, Mao TL (2021). The molecular landscape influencing prognoses of epithelial ovarian cancer. Biomolecules.

